# circFOXO3 facilitated endothelial cell senescence and atherosclerosis through binding to HnRNPK

**DOI:** 10.1016/j.gendis.2025.101517

**Published:** 2025-01-04

**Authors:** Jing-yu Wu, Yu-lan Zhou, Shui-hong Lu, Zhi-peng Yang, Zhao-fu Liao, Dong-liang Liu, Hai-liang Mo, Yi-tuan Xie, Xinguang Liu, Xing-dong Xiong

**Affiliations:** a Dongguan Key Laboratory of Aging and Anti-Aging, Guangdong Provincial Key Laboratory of Medical Immunology and Molecular Diagnostics, The First Dongguan Affiliated Hospital, Guangdong Medical University, Dongguan, Guangdong 523808, China; b School of Medical Technology, Guangdong Medical University, Dongguan, Guangdong 523808, China; c Reproductive Center, The Affiliated Hospital of Guangdong Medical University, Zhanjiang, Guangdong 524001, China; d Department of Neurosurgery, Huizhou First People's Hospital, Huizhou, Guangdong 516003, China

With the development of the social economy and an increasingly aging population, cardiovascular diseases have become a severe threat to public health. Vascular aging is a primary risk factor for the development of cardiovascular diseases and it is associated with the cellular senescence of the vascular endothelium.^1^ Endothelial cell senescence leads to endothelial dysfunction that manifests as impaired endothelium-dependent vasorelaxation and vascular inflammation, ultimately predisposing individuals to atherosclerosis and other cardiovascular complications.^2^ By delaying senescence of vascular endothelial cells, it is possible to reduce or prevent the occurrence and development of atherosclerosis.

Circular RNAs (circRNAs) are a unique class of RNA characterized by a single-stranded, covalently closed, circular structure formed by a back-splicing mechanism. Growing evidence indicates that circRNAs participate in various physiological or pathological processes through multiple functions, including microRNA (miRNA) sponges, transcription and splicing regulation, mRNA traps, translational modulation, and post-translational modifications.^3^ circFOXO3 (hsa_circ_0006404), derived from the human longevity gene *Foxo3* (forkhead box O3), is one of the most studied circRNAs involved in the development of several diseases. Our group previously revealed that the rs12196996 polymorphism in the intronic region of circFOXO3 was associated with susceptibility to coronary heart disease.^4^ However, the role and mechanism of circFOXO3 in endothelial cell senescence have not yet been reported.

To explore the involvement of circFOXO3 in endothelial cell senescence, we examined circFOXO3 expression in young and aged aortas and endothelial cells using quantitative reverse transcription PCR. Our findings revealed that circFOXO3 was highly expressed in aged aortas and endothelial cells (Fig. 1A, B). Since endothelial cell senescence plays a pivotal role in the development of atherosclerosis, we further evaluated whether circFOXO3 was associated with the progression of atherosclerosis. Our data showed that circFOXO3 was significantly up-regulated in atherosclerotic aortic tissue (Fig. 1C). Furthermore, we observed that circFOXO3 was highly expressed in the aorta, particularly in the vascular endothelium, compared with the aortic media (m) and adventitia (a), suggesting that circFOXO3 plays an essential role in endothelial function (Fig. S2A, B). To identify the function of circFOXO3 in endothelial cell senescence, we successfully constructed a lentiviral vector to overexpress circFOXO3 in proliferating endothelial cells. The results revealed that the number of SA-*β*-gal-positive cells increased in endothelial cells following circFOXO3 overexpression, compared with the control group (Fig. 1E, F). Concomitantly, the levels of senescence-associated proteins p16, p53, and p21 also increased (Fig. 1D; Fig. S3), suggesting an elevated state of cellular senescence in cells overexpressing circFOXO3. Moreover, the results of the BrdU incorporation assay and *in vitro* angiogenesis assay showed that circFOXO3 overexpression inhibited endothelial cell proliferation and angiogenesis (Fig. 1G, H).

**Figure 1:**
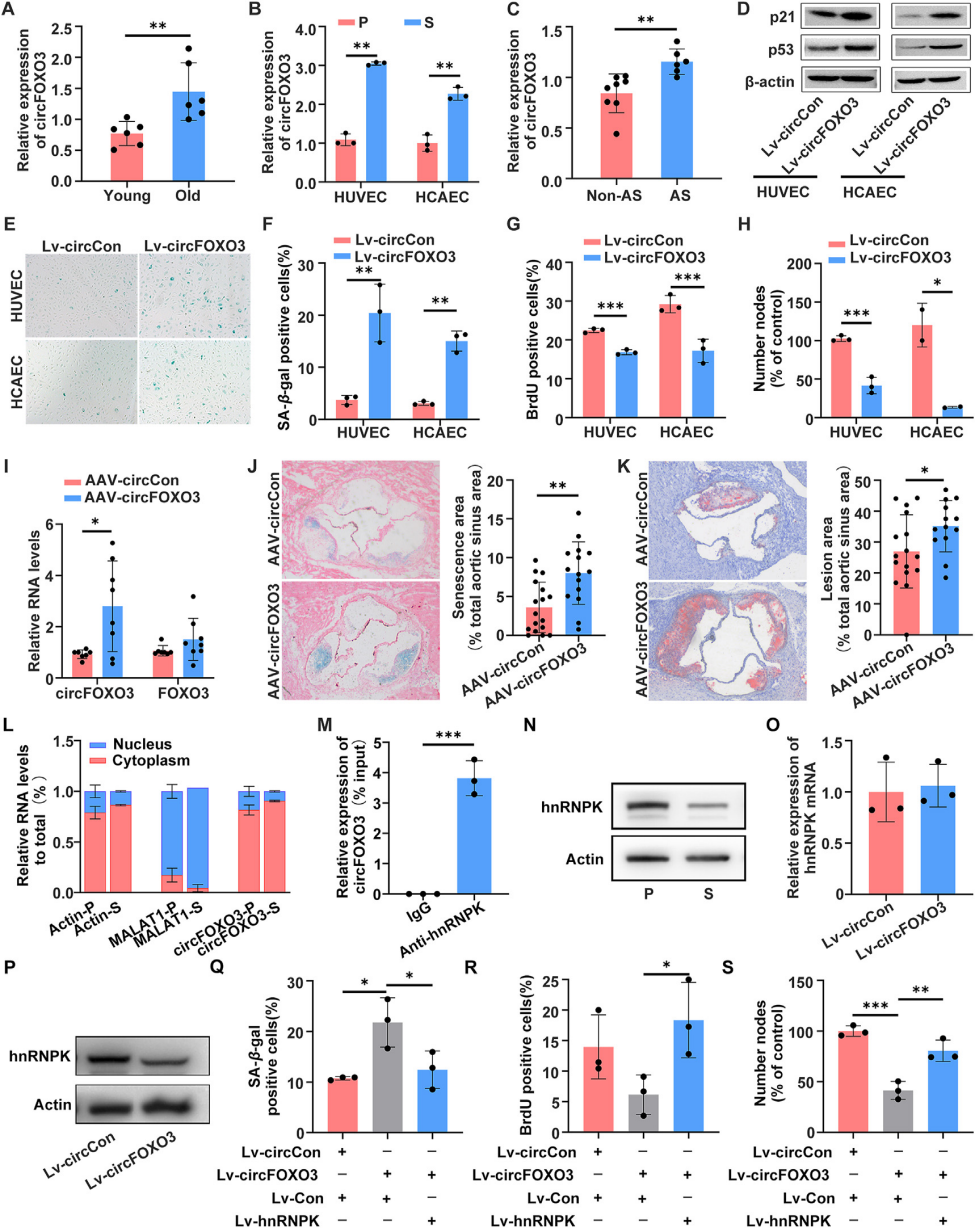
circFOXO3 facilitated endothelial cell senescence and atherosclerosis through binding to hnRNPK. **(A)** Quantitative reverse transcription PCR analysis of circFOXO3 levels in the blood vessels of young (*n* = 6; mean age, 31 years) and old (*n* = 6; mean age, 61 years) individuals. **(B)** Quantitative reverse transcription PCR analysis of circFOXO3 expression in proliferating (P) and senescent (S) endothelial cells. **(C)** Quantitative reverse transcription PCR analysis of circFOXO3 levels in the aorta tissue of atherosclerosis and non-atherosclerotic mice. **(D)** Western blot analysis of p21 and p53 in proliferating endothelial cells after infection with lentiviruses expressing circCon or circFOXO3. **(E)** Representative photographs of the SA-*β*-gal staining of endothelial cells infected with lentiviruses expressing circCon or circFOXO3. **(F)** The SA-*β*-gal positive cells were counted and presented as a percentage of the total cells. **(G)** Statistical summary of BrdU incorporation assays in endothelial cells infected with lentiviruses expressing circCon or circFOXO3. **(H)** Statistical summary of *in vitro* Matrigel assays in endothelial cells infected with lentiviruses expressing circCon or circFOXO3. **(I)** circFOXO3 and linear *FOXO3* expression levels were quantitated in the aortic intima from the AAV-circCon and AAV-circFOXO3 groups. **(J)** SA-*β*-gal activity assays of aortic root sections. **(K)** Lesion areas were detected by oil red O staining of aortic root sections. **(L)** Levels of circFOXO3 in the nuclear and cytoplasmic fractions of human umbilical vein endothelial cells. Actin and MALAT1 were used as positive controls in the cytoplasm and nucleus, respectively. **(M)** RNA immunoprecipitation assay was performed with hnRNPK antibody in human umbilical vein endothelial cells, and then the enrichment of circFOXO3 was detected. **(N)** The protein level of hnRNPK in proliferating (P) and senescent (S) human umbilical vein endothelial cells. **(O, P)** mRNA (O) and protein (P) levels of hnRNPK in human umbilical vein endothelial cells after infection with lentiviruses expressing circCon or circFOXO3. **(Q**–**S)** SA-*β*-gal staining assays (Q), BrdU incorporation assays (R), and *in vitro* Matrigel assays (S) in circFOXO3 lentivirus-infected endothelial cells after infection with lentiviruses expressing Con or hnRNPK. Data are presented as mean ± standard deviation; student's *t*-test or one-way ANOVA; ∗*P* < 0.05, ∗∗*P* < 0.01, ∗∗∗*P* < 0.001.

Based on the *in vitro* results obtained, additional experiments were performed to provide *in vivo* evidence. First, a unique recombinant adeno-associated virus serotype 2/vec (AAV2/vec) was designed to specifically overexpress circFOXO3 in mouse endothelium. *Ldlr*^*−/−*^ mice in each experimental group were administered AAV injections and fed a high-fat diet (Fig. S4A). After 12 weeks, quantitative reverse transcription PCR analysis showed that circFOXO3 expression in the aortic intima was significantly increased in the AAV-circFOXO3 group compared with that in the AAV-circCon (mock vector with no circFOXO3 sequence) group (Fig. 1I; Fig. S4B). There were no differences in plasma lipid profiles between the two groups (Fig. S4C). However, the aortic root sections from the AAV-circFOXO3 group showed higher levels of SA-*β*-gal activity when compared with the AAV-circCon group (Fig. 1J). Meanwhile, the immunofluorescence staining results demonstrated a significant increase in the abundance of p16 in the intimal layer of the aorta in the AAV-circFOXO3 group compared with that in the AAV-circFOXO3 group (Fig. S4D). Quantification of oil red O staining revealed obvious modifications in the atherosclerotic plaque area within the aortic roots (Fig. 1K). Collectively, these findings are consistent with our cytological data, indicating that circFOXO3 significantly contributes to endothelial senescence and the progression of atherosclerotic lesions.

To clarify the mechanism underlying the role of circFOXO3 in endothelial cell senescence, we performed subcellular fractionation to determine the subcellular localization of circFOXO3. Our data showed that circFOXO3 was enriched in the cytoplasm of human umbilical vein endothelial cells (Fig. 1L), prompting us to profile the specific circRNA-RBP complex influenced by circFOXO3. We co-transfected the circFOXO3-MS2 vector with a vector expressing MS2-CP, a protein that specifically binds to the MS2 sequence, and performed MS2-CP-Flag circRNA pull-down assays. The circRNA pull-down products were found to be enriched with the capture protein MS2-CP-Flag, and circFOXO3 was highly abundant in the subsequent capture (Fig. S5A). The pulled-down products were analyzed using tandem mass spectrometry. Among these identified proteins, heterogeneous nuclear ribonucleoprotein K (hnRNPK) was identified as a candidate protein associated with circFOXO3 (Fig. S5B). The interaction between circFOXO3 and hnRNPK was validated using RNA immunoprecipitation (Fig. 1M).

hnRNPK is a multifunctional RNA/DNA-binding protein that is involved in various cellular processes, including chromatin remodeling, RNA processing, and DNA damage response. It has been reported previously that hnRNPK can impact cellular senescence and proliferation through its complex associations with various cellular pathways.^5^ Our data showed that hnRNPK expression was markedly down-regulated in senescent endothelial cells (Fig. 1N). To directly observe the effect of hnRNPK on endothelial cell senescence, two siRNAs were designed to target hnRNPK. Through SA-*β*-Gal activity, we found that down-regulation of hnRNPK promoted endothelial cell senescence (Fig. S6A). Furthermore, hnRNPK knockdown inhibited endothelial cell proliferation and suppressed their ability to differentiate into tubules (Fig. S6B, C). Taken together, these findings suggest that hnRNPK plays a key role in regulating endothelial cell senescence.

In this study, we found that the overexpression of circFOXO3 resulted in a decrease in hnRNPK protein, but not mRNA levels, suggesting that circFOXO3 promotes endothelial cell senescence, probably by affecting the expression of hnRNPK (Fig. 1O, P). To investigate the role of hnRNPK in the regulation of endothelial cell senescence mediated by circFOXO3, we overexpressed hnRNPK in circFOXO3 lentivirus-infected cells. SA-*β*-gal staining displayed that overexpression of circFOXO3 increased SA-*β*-gal-positive cells and the effect could be abrogated by hnRNPK (Fig. 1Q). The data also showed that hnRNPK overexpression could abrogate the suppressive effects of circFOXO3 on endothelial cell proliferation and angiogenic activity, thus inducing a significant increase in BrdU-positive cells and the formation of tube-like structures (Fig. 1R, S). Taken together, hnRNPK overexpression effectively rescued senescence, proliferation, and angiogenic phenotypes induced by circFOXO3 overexpression.

In summary, circFOXO3 is found to be highly expressed in aged arterial intima and atherosclerotic plaques, as well as in senescent vascular endothelial cells. It physically interacts with hnRNPK, and the senescence phenotypes of circFOXO3-overexpressed cells can be effectively rescued by hnRNPK overexpression. These findings provide important insights into the molecular mechanisms underlying endothelial cell senescence and thus offer new avenues for the treatment and prevention of age-related vascular disorders.

## Ethics declaration

This study was approved by the Ethics Committee of the Affiliated Hospital of Guangdong Medical University (approval No. YJYS2020037), and informed consent was obtained from all enrolled participants.

## Funding

The study was supported by grants from the 10.13039/501100001809National Natural Science Foundation of China (No. 82071576, 82300520), the 10.13039/501100021171Guangdong Basic and Applied Basic Research Foundation (China) (No. 2021B1515140058), the Guangdong Province Innovation Team Project for Ordinary Higher Education Institutions (China) (No. 2021KCXTD049), and the Discipline Construction Project of Guangdong Medical University (No. 4SG21008G, 4SG22306P, 4SG24018G).

## CRediT authorship contribution statement

**Jing-yu Wu:** Writing – original draft. **Yu-lan Zhou:** Data curation. **Shui-hong Lu:** Validation. **Zhi-peng Yang:** Formal analysis. **Zhao-fu Liao:** Supervision. **Dong-liang Liu:** Data curation. **Hai-liang Mo:** Investigation. **Yi-tuan Xie:** Investigation. **Xinguang Liu:** Project administration. **Xing-dong Xiong:** Project administration.

## Conflict of interests

The authors declared no competing interests.
