# Supplementary material for: circFOXO3 facilitated endothelial cell senescence and atherosclerosis through binding to HnRNPK
Source: Genes Dis. 2025 Jan 4;12(5):101517. doi: 10.1016/j.gendis.2025.101517 (PMC12166678; doi:10.1016/j.gendis.2025.101517)
Supplement: Multimedia component 1 [file mmc1.docx]

**Experimental procedures**

**Clinical sample and ethics statement**

Human arteries were obtained from young (n = 6; mean age = 31 years) and old (n = 6; mean age = 61 years) individuals who underwent intestinal resection at the Affiliated Hospital of Guangdong Medical University. The perivascular connective tissue was removed on ice within 2 h of collection, and the samples were then placed in cryopreservation tubes preloaded with RNAlater solution and immediately stored in liquid nitrogen. This study was approved by the Ethics Committee of the Affiliated Hospital of Guangdong Medical University (Approval No. YJYS2020037), and informed consent was obtained from all enrolled participants.

**Cell culture**

HUVECs and HCAECs were purchased from ScienCell (Carlsbad, CA, USA). The cells were seeded in endothelial cell medium (ECM) supplemented with 5 % fetal bovine serum and 1% endothelial cell growth supplement (ECGS, ScienCell) in a humidified atmosphere of 5% CO2 at 37 °C.

**RNA isolation and real-time quantitative PCR**

Total RNA was extracted using the TRIzol reagent (Invitrogen) according to the manufacturer’s instructions. The RNA samples were reverse transcribed into cDNA using the PrimeScript RT Reagent Kit (Takara). qPCR was performed on a LightCycler 96 System (Roche) using the TB Green Premix Ex Taq Kit (Takara).  Each experiment was replicated thrice, and the relative expression was calculated utilizing the 2^-ΔΔCt^ method. *β*‐actin was selected as internal reference. The primers used in this study are listed in Table.

**Western blot assay**

The cells were lysed in RIPA buffer (Beyotime) containing 1 mM PMSF. The cell lysates were separated by 12.5% SDS-PAGE gels and then transferred onto polyvinylidene fluoride (PVDF) membranes (Millipore, Billerica, MA, USA). The membranes were blocked in 5% milk in TBST and soaked in primary antibodies overnight at 4°C. The following day, after incubation with the appropriate horseradish peroxidase-conjugated secondary antibody, bands were detected using the Immobilon Western Chemiluminescent HRP Substrate (Millipore Sigma).

**Senescence-associated *β* -galactosidase staining**

For the senescence-associated staining, the Senescence *β*‐Galactosidase Staining Kit (Beyotime) was used according to the manufacturer’s instructions. Images were taken at 100× magnification for six randomly selected microscopic fields and positively stained (blue) as well as total cells were counted manually to calculate the percentage of positively stained cells.

**BrdU incorporation assay**

BrdU incorporation (cell proliferation) was measured using BrdU immunostaining. 40 μM BrdU was added to normal medium and incubated for 1 h for cells to take up BrdU. Subsequently, cells were fixed in 4% paraformaldehyde, treated with 0.05% trypsin, permeabilized, and blocked with 3% BSA. BrdU was detected using an anti-BrdU primary antibody (Cell Signaling Technology) and visualized using an Alexa Fluor 488/555‐conjugated secondary antibody (Cell Signaling Technology). An Invitrogen EVOS FL Auto Cell Imaging System (Thermo Fisher Scientific) was used to capture images at 100× magnification.

***In vitro* angiogenesis assay**

96 well plates were pre-coated with 100 μl Matrigel (Corning) at 37 °C for 1 h, following this, 1×10^4^ cells were seeded into each well. After culturing for 4–6 h at 37℃, tubule formation was imaged at 100× magnification and analyzed using the Image J programme.

**Immunofluorescence**

4% paraformaldehyde (Biosharp) was used to fix cultured cells or aortic root sections from mice for 15 min. The samples were blocked with 5% normal goat serum (Vector) with 0.1% Triton X-100 (Solarbio) in PBS for 60 min at room temperature, and then incubated with primary antibodies overnight at 4 °C followed by the appropriate secondary fluorescently labeled antibodies (Abcam) for one hour at room temperature. The nuclei were counterstained with DAPI. Images were acquired using a confocal microscope (Leica).

**CircRNA pull-down assay and mass spectrometry analysis**

The RNA-binding proteins (RBPs) associated with circFOXO3 were determined by using a circRNA pull-down assay with MS2-capturing protein (MS2-CP). Briefly, two overexpression vectors, one carrying circFOXO3-MS2 and the other carrying MS2-CP-Flag, were constructed (Geneseed, China) and then co-transfected into 293T cells. Following the specific interaction between MS2-tagged circFOXO3 and MS2-CP, the complex was precipitated using an anti-Flag antibody. Lysate from 293T cells lacking the MS2 flagging system served as a control. The captured complexes were identified using real-time quantitative PCR (RT-qPCR) and Western blotting. Finally, both the circFOXO3 pull-down complex and the control were subjected to mass spectrometry analysis.

**RNA-binding protein immunoprecipitation（RIP）**

The RIP assay was performed as described ^1^. Briefly, cells were collected in cold PBS and lysed in polysome lysis buffer [100 mM KCl, 10 mM HEPES-NaOH (pH 7.0), 0.5% NP40, 5 mM MgCl2, 1 mM DTT, 200 U/ml RNase inhibitors, and protease inhibitor cocktail]. The cell lysates were sonicated for chromatin and RNA fragmentation. Proteins were incubated with Pierce Protein A/G Magnetic Beads (88803; Thermo Fisher Scientific) pre-coated with mouse anti-hnRNPK antibodies (no. sc-28380, Santa Cruz Biotechnology), or normal mouse IgG (no. sc-2025; Santa Cruz Biotechnology) overnight at 4°C. 1/10 of the supernatant was set aside for input before incubation. After 6 washes with NT-2 buffer [50 mM Tris-HCl (pH 7.4), 1 mM MgCl2, 150 mM NaCl, and 0.05% NP-40], the proteins were then digested by incubating with Proteinase K digestion buffer [NT-2 buffer supplemented with 1 % sodium dodecyl sulfate (SDS), 1.2 mg/ml Proteinase K] at 55°C for 30 minutes. Immunoprecipitated RNA and input RNA were isolated and purified using TRIzol reagent (Invitrogen). The expression of the co-precipitated RNA was quantified using RT-qPCR.

**Animal experiments**

To avoid the normal fluctuation of estrogen affecting the results, six-week-old male low-density lipoprotein receptor knockout (*Ldlr*−/−) mice were purchased from GemPharmatech (Nanjing, Jiangsu, China) and then they were randomized into 2 groups. All of the mice were housed at 21°C and 60% humidity with an artificial 12:12 h light–dark cycle and were fed with ad libitum standard chow and water. Recombinant adeno-associated virus serotype 2/VEC (AAV2/VEC) carrying the mouse circFOXO3 gene and empty vector with the mouse endothelial-specific promoter Tie (AAV2/VEC-Tie-circFOXO3 and AAV2/VEC-Tie-empty) were constructed and synthesized by Hanheng Biotechnology Co., Ltd. (Shanghai, China). AAV2/VEC-Tie-empty cells were used as negative controls. At eight weeks of age, mice were injected with AAV (1.3 × 10^11^ viral particles for each mouse) via the tail vein and placed on a high-fat diet. Twelve weeks after injection, the mice were sacrificed and their hearts and aortae were carefully isolated. The hearts were embedded in OCT (Sakura), and serial sections of 8 µm in thickness were collected from the aortic roots where the aortic valve appears.

**Intimal RNA isolation from aorta tissue**

The intimal RNA was isolated as previously described ^2^. Aortae were flushed with 100 μl TRIzol by 29-gauge insulin syringe to elute endothelial RNA, which was used to determine the gene expression in vascular endothelial cells. After flushing with TRIzol, the aorta was used to prepare RNA from the media and adventitia.

**Atherosclerotic lesion analysis**

The severity of atherosclerotic lesions was assessed by analyzing lesions in the aortic root (heart sinus). For analyzing the aortic root plaque lesions, 8 µm frozen sections of the aortic root were prepared. Sections were stained with Oil Red O, counterstained with Mayer’s hematoxylin, photographed, and then digitized for lesion analysis. The results for each group are expressed as a percentage of the total aortic area containing the lesions.

**Statistical analysis**

Statistical analyses were performed using GraphPad Prism 8.0 (GraphPad Software, San Diego, CA, USA) and SPSS Statistics (version 20.0; IBM, Armonk, NY, USA). The experiments were repeated at least 3 times. All data are mean ± standard deviation (SD), the Student’ s *t* test and one-way analysis of variance (ANOVA) were applied for calculating statistical probabilities. Statistical significance was set at *p* values less than 0.05 were considered significant.

**Methods references**

1. Gagliardi M, Matarazzo MR. RIP: RNA Immunoprecipitation. *Methods Mol Biol*. 2016;1480:73-86. doi:10.1007/978-1-4939-6380-5_7

2. Wu W-P, Zhou M-Y, Liu D-L, et al. circGNAQ, a circular RNA enriched in vascular endothelium, inhibits endothelial cell senescence and atherosclerosis progression. *Mol Ther Nucleic Acids*. 2021;26:374-387. doi:10.1016/j.omtn.2021.07.020

**Table S1 Primers and oligoes used in this study**

|  | Name | | Sequence (5'--3') |
| --- | --- | --- | --- |
| Primers | ACTIN | F | AGATGACCCAGATCATGTTTGAG |
|  |  | R | AGGGCATACCCCTCGTAGAT |
|  | CircFOXO3 | F | ATGGATGCTGATGGGTTGGA |
|  |  | R | AGGTTGTGCCGGATGGAGTT |
|  | FOXO3 | F | TCTACGAGTGGATGGTGCGTTG |
|  |  | R | CTCTTGCCAGTTCCCTCATTCTG |
|  | HnRNPK | F | CAATGGTGAATTTGGTAAACGCC |
|  |  | R | GTAGTCTGTACGGAGAGCCTTA |
|  | mouse-circFOXO3 | F | GGCCTCATCTCAAAGCTGG |
|  |  | R | CTTGCCCGTGCCTTCATT |
|  | mouse-FOXO3 | F | GGGGAGTTTGGTCAATCAGA |
|  |  | R | GAGTCACTCAAGCCCATGTTG |
| RNA oligos | si-hnRNPK-1 | Sense | ATCCCAATTTTTACGATGA |
|  | si-hnRNPK-2 | Sense | GCATAAAGATCATCCTTGA |
|  | si-NC | Sense | AACACGTCTATACGC |

**Figure S1**

**
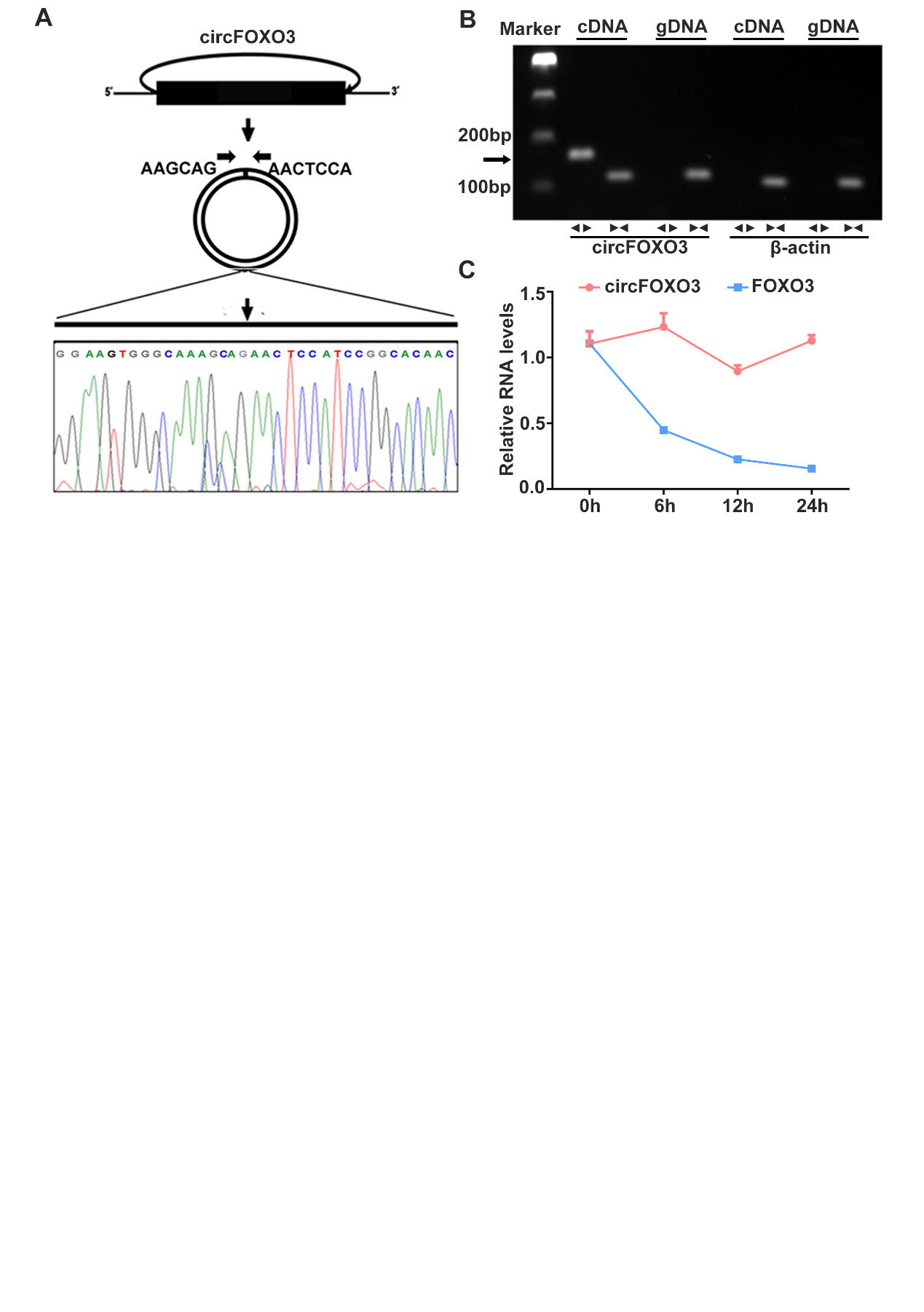
**

**Figure S1** **Characterization of circFOXO3.** (A) Schematic illustration of circFOXO3 formation through the circularization of exons 2 in *FOXO3* gene. Black arrow represents the back-splice junction confirmed by Sanger sequencing. (B) RT-qPCR or PCR assay indicating the detection of circFOXO3 using the divergent and convergent primers from cDNA or genomic DNA (gDNA) of HUVECs. β-actin was used as the negative control. (C) CircFOXO3 and linear FOXO3 expression levels were detected after actinomycin D treatment in HUVECs. Data are presented as mean ± SD.

**Figure S2**

**
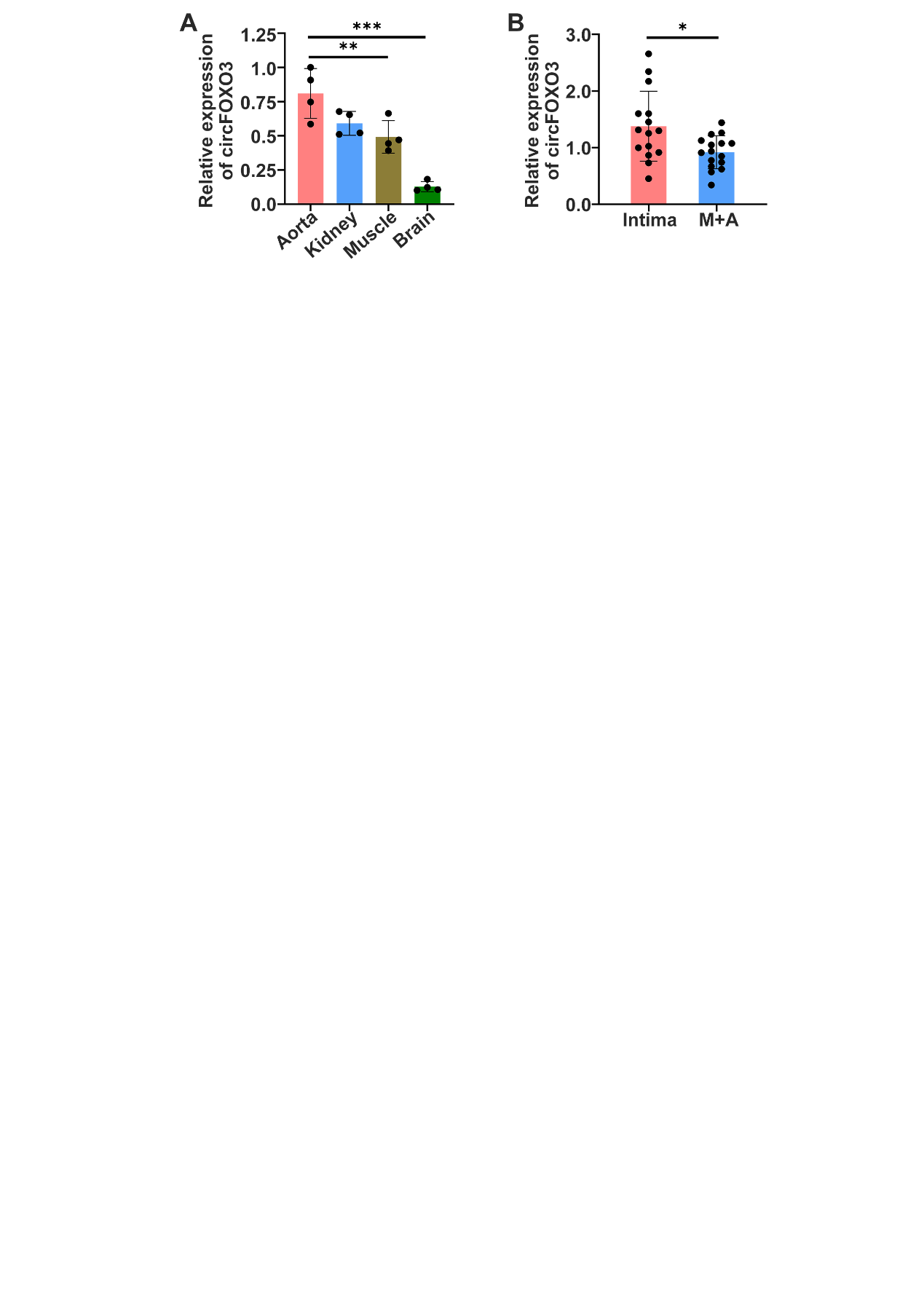
**

**Figure S2. The expression of circFOXO3 in the various tissues.** (A) RT-qPCR analysis of circFOXO3 levels in the various tissues of C57BL/6 mice. (B) circFOXO3 expression was detected by RT-qPCR in the aortic intima from 8-week-old C57BL/6 mice (n = 4; each sample represents RNA pooled from two mice). RNA from the media (m) and adventitia (a) of these arteries served as the control. Data are presented as mean ± SD; * *p* < 0.05, ** *p* < 0.01, *** *p* < 0.001 by one-way ANOVA or Student’s *t*-test.

**Figure S3**

**
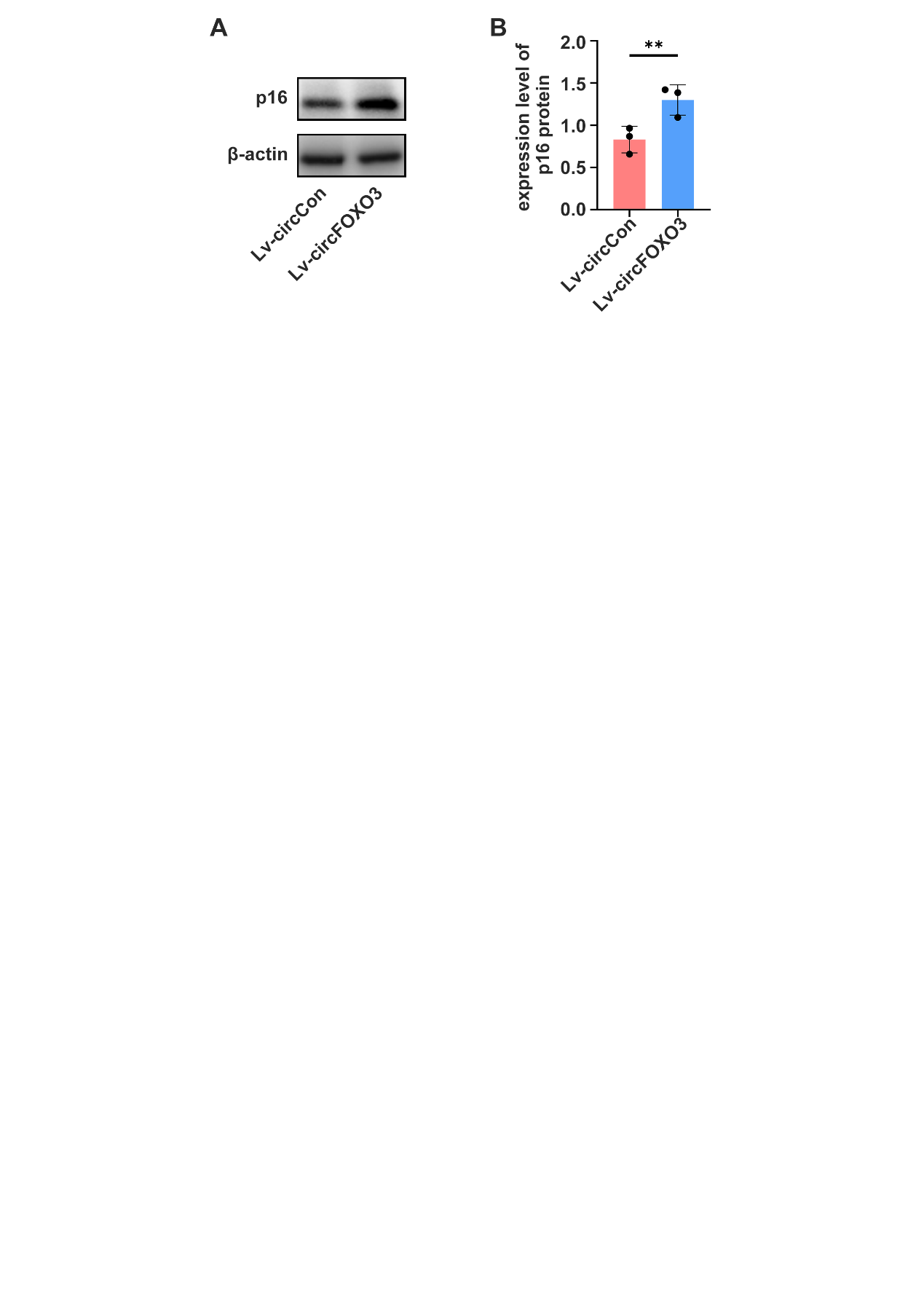
**

**Figure S3. The effects of circFOXO3 overexpression on p16 protein level in endothelial cells.** (A, B) Western blot analysis of p16 in proliferating HUVEC after infection with lentiviruses expressing circCon or circFOXO3. Data are presented as mean ± SD; ** *p* < 0.01 by Student’s *t*-test.

**Figure S4**

**
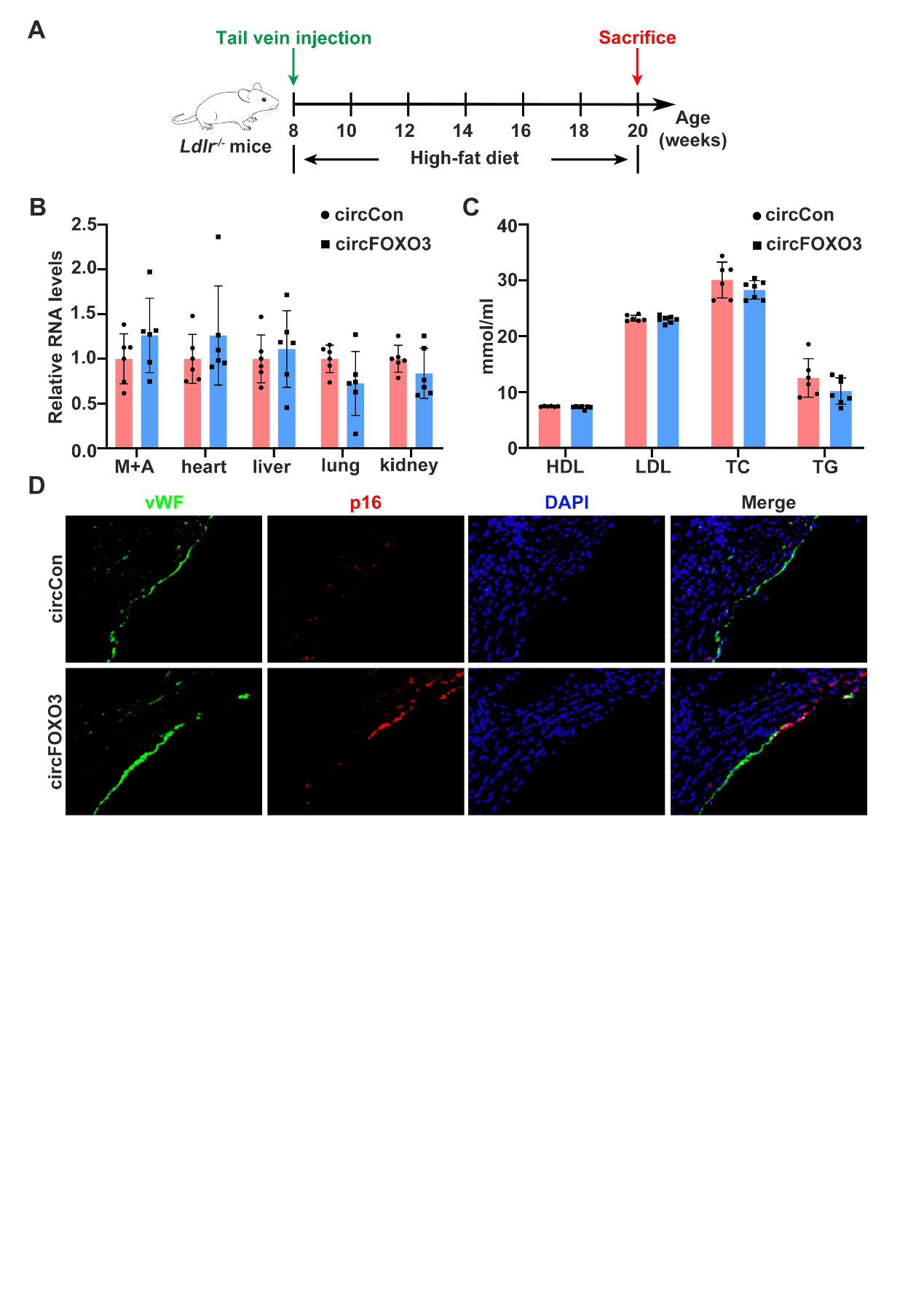
**

**Figure S4. The effects of circFOXO3 overexpression in *Ldlr*^-/-^ mice.** (A) Schematic overview of the animal experiment. (B) CircFOXO3 and linear FOXO3 expression levels were quantitated in the aortic intima from the AAV-circCon and AAV-circFOXO3 groups. (C) Plasma levels of high-density lipoprotein (HDL), low-density lipoprotein (LDL), total cholesterol (TC) and triglyceride (TG) from the AAV-circCon and AAV-circFOXO3 groups. (D) Immunofluorescence staining of p16 and vWF in the atherosclerotic lesions. Data are presented as mean ± SD.

**Figure S****5**

**
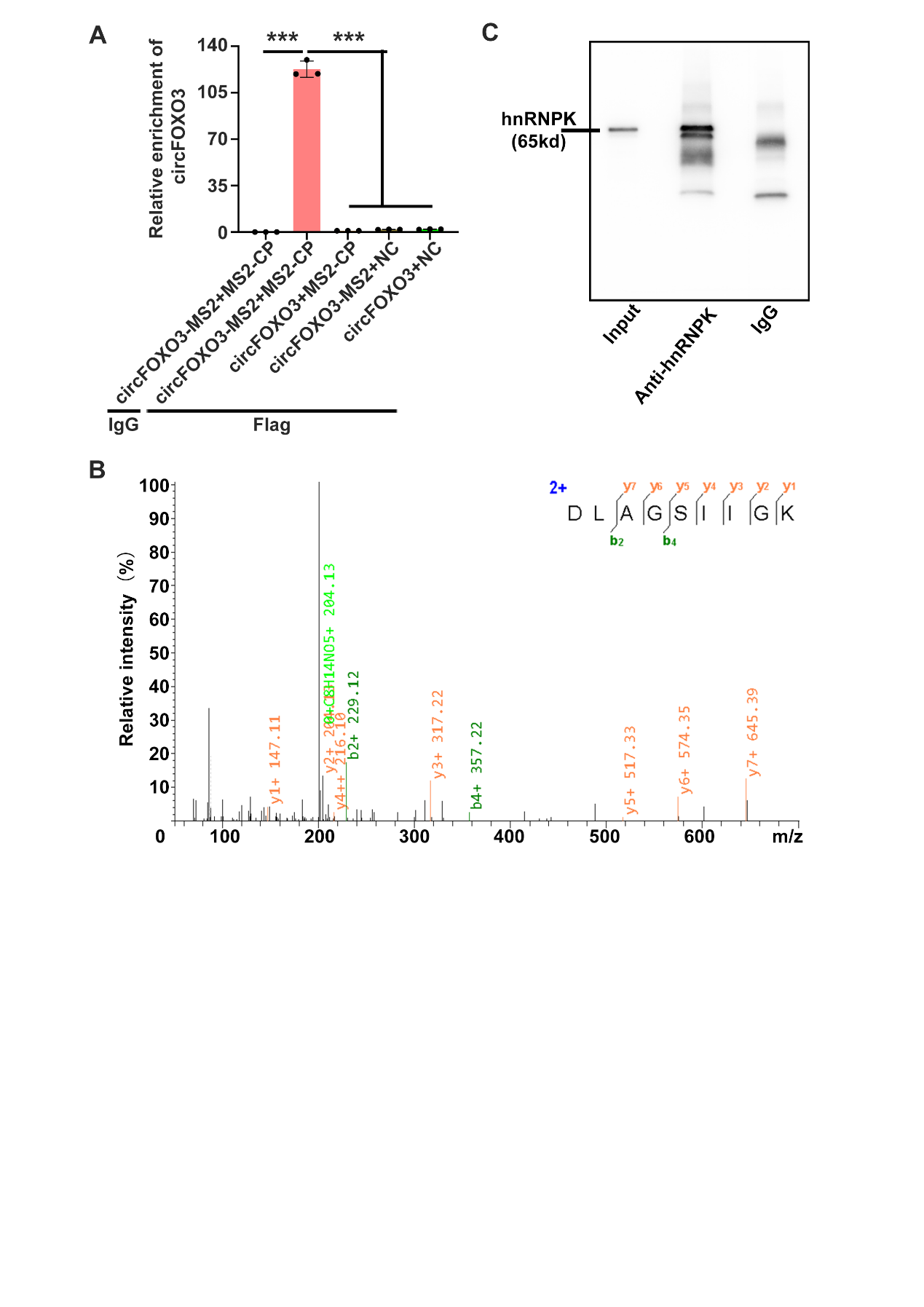
**

**Figure S5. The interaction between circFOXO3 and hnRNPK.** (A) The enrichment of circFOXO3 in complex with MS2-CP-Flag as measured by RT-qPCR in the circRNA-pull down assay. (B) circRNA pull-down assay by MS2-labeled circFOXO3 and unlabeled circFOXO3 followed by mass spectrometry of pull-down product. The MS spectra of the peptides used for hnRNPK identification were shown. (C) HnRNPK was immunoprecipitated in RIP assay using an anti-hnRNPK antibody. Data are presented as mean ± SD; ****p* < 0.001 by one-way ANOVA.

**Figure S6**

**
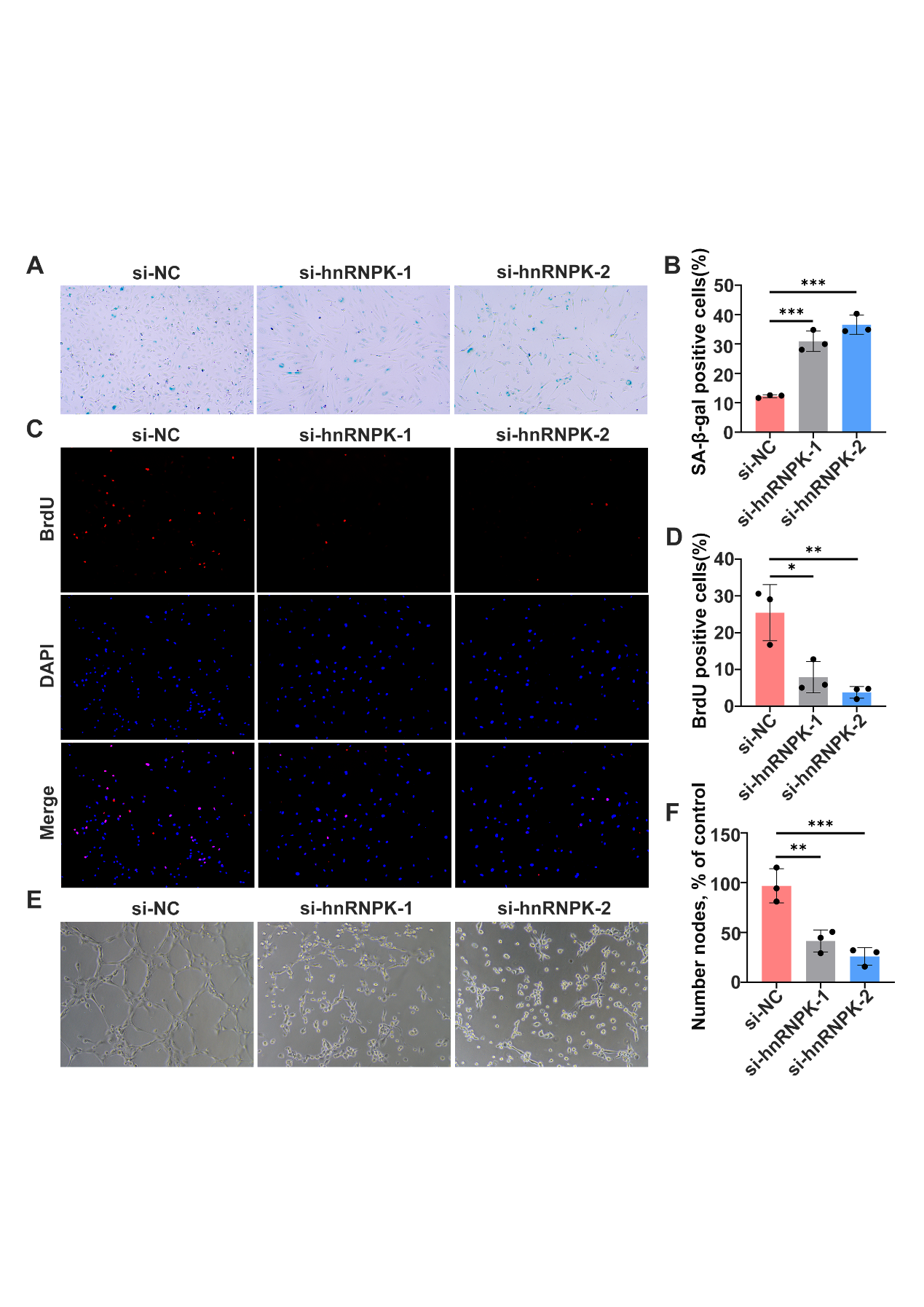
**

**Figure S6. The effects of hnRNPK knockdown in endothelial cell senescence.** Statistical summary of (A, B) SA-*β*-gal staining, (C, D) BrdU incorporation assays and (E, F) *in vitro* Matrigel assays in si-NC-, si-hnRNPK-1-, and si-hnRNPK-2-transfected endothelial cells. Data are presented as mean ± SD; **p* < 0.05, ***p* < 0.01, ****p* < 0.001 by one-way ANOVA.
